# Whole-Genome Sequence of the First Sequence Type 27 *Brucella ceti* Strain Isolated from European Waters

**DOI:** 10.1128/genomeA.00988-17

**Published:** 2017-09-14

**Authors:** Sanja Duvnjak, Silvio Špičić, Darja Kušar, Bojan Papić, Irena Reil, Maja Zdelar-Tuk, Željko Pavlinec, Martina Đuras, Tomislav Gomerčić, Rene S. Hendriksen, Željko Cvetnić

**Affiliations:** a National Reference Laboratory for Brucellosis, Laboratory for Bacterial Zoonosis and Molecular Diagnostics of Bacterial Diseases, Croatian Veterinary Institute, Zagreb, Croatia; b Institute of Microbiology and Parasitology, Veterinary Faculty, University of Ljubljana, Ljubljana, Slovenia; c Faculty of Veterinary Medicine, University of Zagreb, Zagreb, Croatia; d Technical University of Denmark, National Food Institute, Søltofts Plads, Lyngby, Denmark

## Abstract

*Brucella* spp. that cause marine brucellosis are becoming more important, as the disease appears to be more widespread than originally thought. Here, we report a whole and annotated genome sequence of *Brucella ceti* CRO350, a sequence type 27 strain isolated from a bottlenose dolphin carcass found in the Croatian part of the northern Adriatic Sea.

## GENOME ANNOUNCEMENT

*Brucella* spp. are known as very important zoonosis-causing terrestrial pathogens, and for the last two decades they have been known to cause brucellosis in marine mammals. Marine *Brucella* spp. have been classified into two groups. Isolates from cetaceans are classified as *B. ceti*, while isolates from pinnipeds are classified as *B. pinnipedialis* (1, 2). However, the division is not perfect, and cross-species infections, as well as zoonotic transmission, are possible (3). Mediterranean *B. ceti* strains have been isolated from striped dolphins in the Tyrrhenian Sea (4) and Ionian Sea (5), as well as from two striped dolphins and one bottlenose dolphin in the Spanish Mediterranean (6). All of these *B. ceti* strains belong to sequence type 26 (ST26) based on multilocus sequence typing (MLST) (5).

*Brucella ceti* strain CRO350 was isolated from a bottlenose dolphin (*Tursiops truncatus*) carcass found in the Croatian part of the northern Adriatic Sea during the summer of 2015 (7). Available molecular techniques identified this strain as a marine *Brucella* sp.; multilocus variable-number tandem-repeat analysis (MLVA) genotyping identified this strain closer to *B. pinnipedialis*, and MLST identified it as *B. ceti* ST27. To our knowledge, this is the first evidence of an ST27 strain in the Adriatic Sea and in European waters in general. The first ST27 strain was isolated from an aborted bottlenose dolphin fetus at an aquarium in San Diego, California, USA (strain F5/99) (3), and, subsequently, in the fetuses and neonates of bottlenose dolphins in South Carolina, USA (8). So far, ST27 appears to be the only sequence type known to have the ability to infect humans naturally (3, 9).

Not many *B. ceti* whole-genome sequences are currently available. Only one ST27 *B. ceti* strain is currently available publicly (10). Here, we report a whole-genome sequence of *B. ceti* strain CRO350. Genomic DNA was extracted using a QIAamp DNA minikit on a QIAcube (Qiagen, Germany). DNA concentrations were determined using the Qubit double-stranded DNA (dsDNA) BR assay kit (Invitrogen, Thermo Fisher Scientific, USA). The genomic DNA was prepared for Illumina (Illumina, USA) paired-end sequencing using the Illumina NexteraXT guide (no. 150319425031942), following protocol revision C.

Paired-end reads were trimmed for adapter sequences and low-quality bases using Cutadapt version 1.13 (11), and a minimum Phred quality score threshold of 20 was established. Reads were assembled using SPAdes version 3.9.1 (parameters, –k 21, 33, 55, 77, and 99; –careful; and –cov-cutoff 3) (12). The assembly yielded 76 contigs, which were reordered using Mauve Contig Mover implemented in Mauve version 2.4.0 (13). The length of the assembled nucleotides (nt) was 3,365,450 nt, the *N*_50_ value was 124,418 nt, and the G+C content was 57.2%. Average coverage was 30.6×. Prokka version 1.12 (14) was used for genome annotation; the genome consisted of 1 transfer-messenger RNA, 3,131 coding sequences, 3 rRNAs, and 47 tRNAs.

### Accession number(s).

This whole-genome shotgun project has been deposited at DDBJ/ENA/GenBank under the accession number NKHE00000000. The version described in this paper is the first version, NKHE01000000.
